# The Use of Essential Oil and Hydrosol Extracted from *Satureja hellenica* for the Control of *Meloidogyne incognita* and *M. javanica*

**DOI:** 10.3390/plants9070856

**Published:** 2020-07-07

**Authors:** Iro Pardavella, Eleni Nasiou, Dimitra Daferera, Panayiotis Trigas, Ioannis Giannakou

**Affiliations:** 1 Laboratory of Agricultural Zoology and Entomology, Department of Science of Crop Production, Agricultural University of Athens, Iera Odos 75, 11855 Athens, Greece; iro.pardavella@gmail.com (I.P.); lenanasiou@gmail.com (E.N.); 2 Laboratory of Chemistry, Department of Food Science and Human Nutrition, Agricultural University of Athens, Iera Odos 75, 11855 Athens, Greece; daferera@aua.gr; 3 Laboratory of Systematic Botany, Department of Science of Crop Production, Agricultural University of Athens, Iera Odos 75, 11855 Athens, Greece; trigas@aua.gr

**Keywords:** *Meloidogyne incognita*, *M. javanica*, *Cuminum cyminum*, essential oil, hydrosol, root-knot management

## Abstract

Essential oil (EO) and hydrosol (HL) isolated from an indigenous plant species *Satureja hellenica* were evaluated against *Meloidogyne incognita* and *M. javanica*. Particularly, the activity of extracts on a second stage juvenile’s (J2s) motility, the hatching of J2s from eggs, egg differentiation and the effect on J2s in soil were tested. A paralysis of 100% of the J2s of both species was recorded after 96 h of immersion in the essential oil, at a dose of 2000 μL/L. At the same dose, the percentage of paralyzed J2s after 48 h of immersion was more than 80%, for both *Meloidogyne* species. The use of hydrosol has shown encouraging results only in the dilution of 50%, where for both *Meloidogyne* species tested, the percentage of paralyzed J2s was more than 70% after 48 h of immersion, while the percentage was increased to 90% after 96 h of immersion. Egg differentiation was ceased after immersion, either in EO or HL. However, this decrease in egg differentiation was evident only at higher concentrations of EO and at the highest HL dilution (0.5 *v*/*v*). The hatching of *M. incognita* J2s was decreasing as the dose was increasing. The lowest numbers of hatched J2s were recorded at the doses of 2000 and 4000 μL/L. A clear reduction in *M. javanica* J2s hatching was observed as the dose was increased to 250 μL/L, a fact constantly observed as the dose was increasing up to 4000 μL/L. Lower numbers of nematodes were recorded in roots grown in infested soil after the application of EO or HL at the highest doses. The EO of *S. hellenica* is characterized by the presence of *p*-cymene (27.46%) and carvacrol (23.25%), and in a lesser extent of other constituents, such as borneol (6.79%), carvacrol methylether (6.77%), *γ*-terpinene (4.63%) and 4-terpineol (3.65%). Carvacrol was the major constituent found in the HL (50.12%), followed by borneol and 4-terpineol (20.42 and 6.72%, respectively).

## 1. Introduction

Plant parasitic nematodes are the most destructive group of plant pathogens worldwide, and their control is extremely challenging [1]. Many crops all over the world can be infested by nematodes, creating yield losses of over a billion Euros annually [2]. In the recent past years, a lot of nematicides have been widely used to suppress the root-knot nematodes (RKN) populations in vegetable production. However, some nematicides, such as methyl bromide, have been banned, due to their adverse effect on the environment. Nematodes management is based on cultural practices, crop rotation, resistant cultivars or root stocks and the use of a limited number of synthetic chemical nematicides. However, for the ones that are still used, their repetitive application reduced their persistence and efficacy due to enhanced degradation [3,4]. A lot of research has been undertaken to find new molecules which are effective against plant parasitic nematodes [5,6,7,8,9,10]. Plant derived phytochemicals can be used as pesticides themselves, or they can serve as model compounds for the agrochemical industry [6]. Towards this, plant essential oils (EOs) and their components have been repeatedly tested against sedentary and migratory endoparasitic nematodes [6]. Plants produce a set of volatile organic compounds as a defense mechanism against damage by pests and pathogens. EOs are natural volatile substances extracted from a wide range of plants belonging to different botanical families. Essential oils consist of a mixture of terpenoids [11]. Their chemical composition is not stable amongst the plant species and varieties, or even amongst the phenological stages of the plant. Although EOs have long been investigated, not much attention has been paid to hydrosols, which are by-products derived during hydrodistillation. Hydrosols derived from three Lamiaceae species showed an important adverse effect on the settling behavior of *Myzus persicae* [12]. Traka et al. [13] reported that hydrosols derived from *Ocinum basilicum* and *Ruta chalepensis* presented an important effect on the mortality of *Aphis gossypii* and *Tetranychus urticae*. Terpenes constituent of EOs are responsible for the characteristic aroma and odor of each plant, because of the volatile compounds that they contain. The presence of volatile terpenes provides EOs with antimicrobial, antifungal [14], antioxidant [15], insecticidal [16,17] and nematicidal properties [18]. A large number of EOs and their components have been found to have nematicidal activity against pinewood (*Bursaphelenchus xylophilus*) and root-knot nematodes (*Meloidogyne* spp.) [6,19,20].

The objective of this study was the evaluation of the nematicidal activity of the essential oil and hydrosol obtained from an indigenous species *Satureja hellenica*, and their correlation to the chemical composition.

## 2. Results

### 2.1. Chemical Composition of Essential Oil and Hydrosol

The data in Table 1 show the identified compounds in order of their elution from the TR-5MS capillary column, the relative retention indices (RRI), and the compounds’ relative percentages in the total composition of the EO and hydrosol. Thirty-two constituents were identified from the EO, while 18 constituents were recorded in the hydrosol. The EO is characterized by the presence of *p*-cymene (27.46%) and carvacrol (23.25%), and in a lesser extent of other constituents such as borneol (6.79%), carvacrol methylether (6.77%), *γ*-terpinene (4.63%) and 4-terpineol (3.65%). Carvacrol was the major constituent found in the hydrosol (50.12%) followed by borneol and 4-terpineol (20.42 and 6.72%, respectively).

### 2.2. Nematicidal Effect of Essential Oil and Hydrosol

The numbers of dead *M. incognita* and *M. javanica* second stage juveniles (J2s), as affected by the different doses of the essential oil, are presented in Table 2 and Table 3, respectively. The percentage of dead J2s increased as the exposure time increased. The essential oil killed 100% of the J2s of both species after 96 h of immersion at a dose of 2000 μL/L. At the same dose, the percentage of dead J2s after 48 h of immersion was more than 80%, for both *Meloidogyne* species.

The percentage of dead *M. incognita* and *M. javanica* J2s, as affected by the different dilutions of the hydrosol (HL), is presented in Table 4 and Table 5, respectively. The hydrosol gave encouraging results only in the dilution of 0.5 *v*/*v*, where for both species, the percentage of dead J2s was more than 70% after 48 h of immersion. At the same dilution, the percentage was more than 90% after 96 h of immersion for both *Meloidogyne* species. In both nematode species, lower HL concentrations of *S. hellenica* did not affect the motility of juveniles after immersion, either for 24 or 48 h. However, a decrease of J2s motility was observed after 96 h immersion amongst the low concentrations of the HL and the control for both *Meloidogyne* species.

### 2.3. Effect of EO and HL on Egg Differentiation

**Figure 1:**
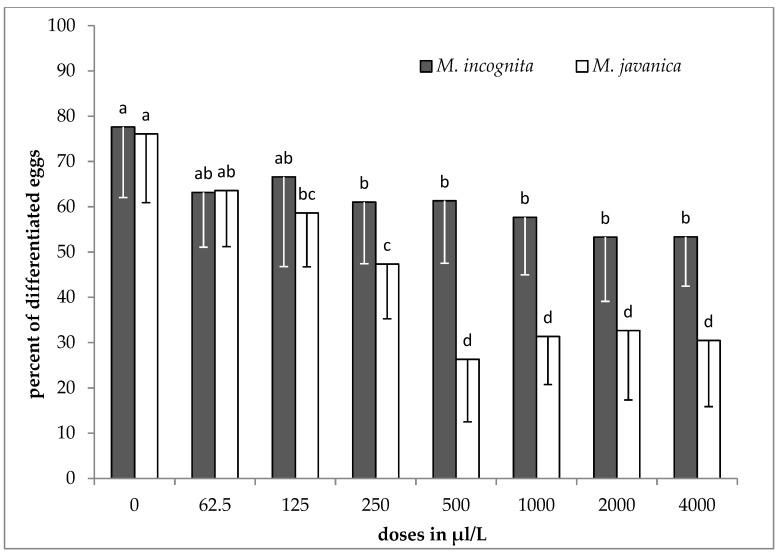
Effect of essential oil on the differentiation of *M. incognita* and *M. javanica* eggs after immersion in test solutions, at the doses of 0, 62.5, 125, 250, 500, 1000, 2000 and 4000 μL/L for 28 days. Bars with the same color followed by the same letter are not significantly different. Error bars represent the standard deviation of mean.

The results of the effect of the essential oil on egg differentiation, as means of the two experiments, are shown in Figure 1 for both species. No significant differences were recorded among the different concentrations of the EO for *M. incognita*. However, by increasing the concentration to 250 μL/L, a significant reduction of the differentiated eggs was observed compared to the control. A different pattern was observed when *M. javanica* was used. The essential oil substantially inhibited eggs differentiation when tested at doses of 500, 1000, 2000 and 4000 μL/L. Egg differentiation was also inhibited when undifferentiated eggs were treated with lower doses of EO (125 and 250 μL/L).

**Figure 2:**
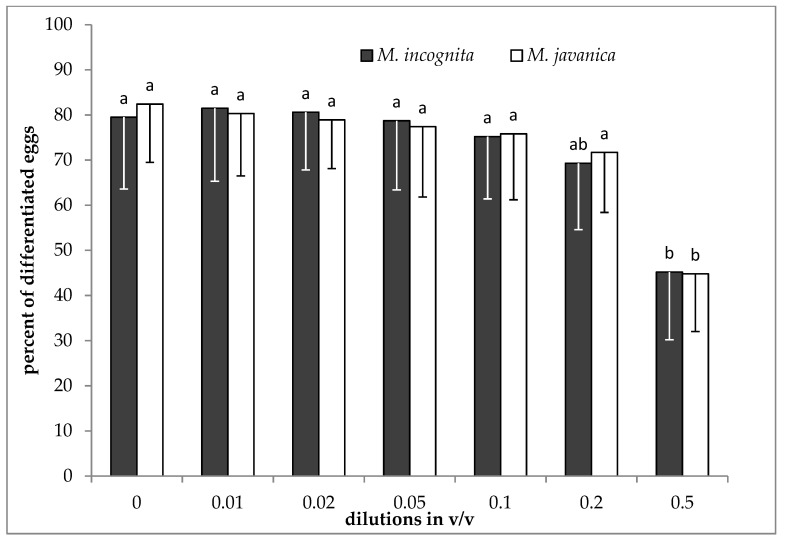
Effect of hydrosol on the differentiation of *M. incognita* and *M. javanica* eggs after immersion in test solutions at the doses of 0, 0.01, 0.02, 0.05, 0.1, 0.2 and 0.5 *v*/*v*, for 28 days. Bars with the same color followed by the same letter are not significantly different. Error bars represent the standard deviation of mean.

No significant differences were recorded among the different doses of *S. hellenica* HL for both species tested, except that of 0.5 *v*/*v*, which was significantly different to the control, and also to the lower doses (Figure 2).

### 2.4. Hatching Inhibition as Affected by the Presence of EO and HL

**Figure 3:**
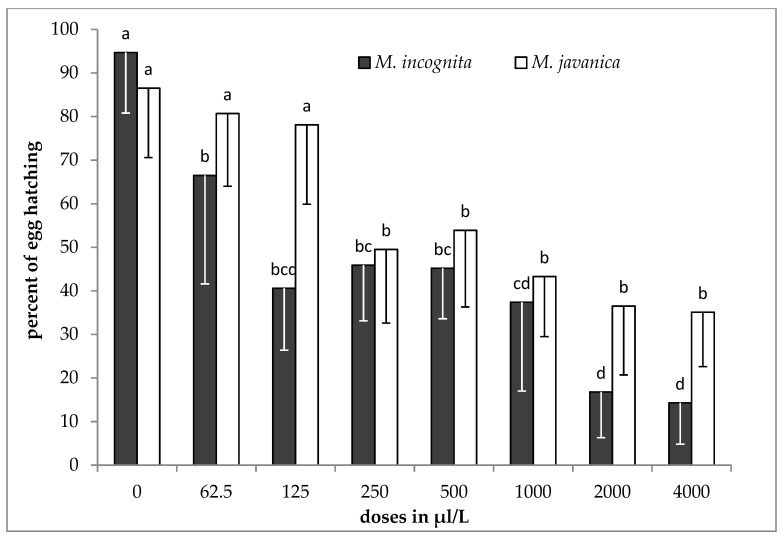
Effect of essential oil on hatching of *M. incognita* and *M. javanica* J2s after immersion in test solutions at the doses of 0, 62.5, 125, 250, 500, 1000 and 2000 μL/L for 35 days. Bars with the same color followed by the same letter are not significantly different. Error bars represent the standard deviation of mean.

The results of the effect of the essential oil on hatching inhibition, as means of the two experiments, are presented in Figure 3. The effect of the essential oil on hatching of J2s was related to different doses after 35 days exposure for both species. The hatching of *M. incognita* J2s was decreasing as the dose was increasing. The lowest numbers of hatched J2s were recorded at the doses of 2000 and 4000 μL/L. A clear reduction in *M. javanica* juveniles hatching was observed, as the dose was increased to 250 μL/L, a fact constantly observed as the dose was increasing up to 4000 μL/L.

**Figure 4:**
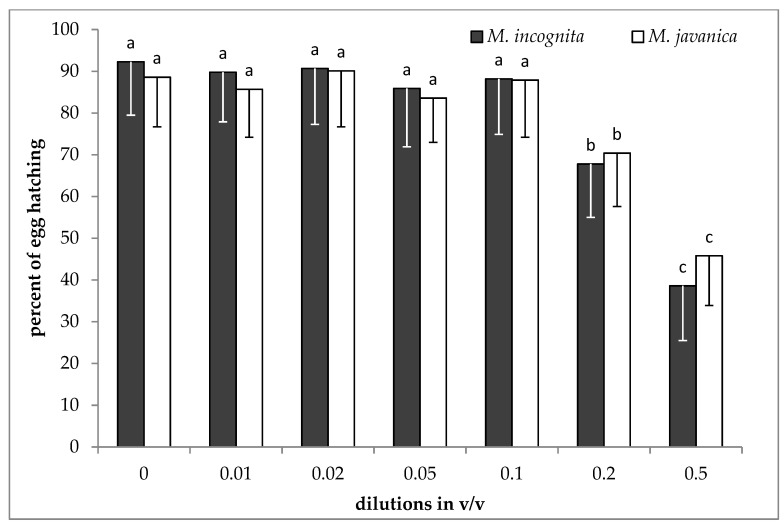
Effect of hydrosol on hatching of *M. incognita* and *M. javanica* J2s after immersion in test solutions, at the doses of 0, 0.01, 0.02, 0.05, 0.1, 0.2 and 0.5 *v*/*v* for 28 days. Bars with the same color followed by the same letter are not significantly different. Error bars represent the standard deviation of mean.

No significant differences were recorded for both species among the control and the doses up to 0.1 *v*/*v* of hydrosol to water (Figure 4). A reduction in J2s hatching was observed when the dose of hydrosol was increased to 0.2 *v*/*v*, and a further reduction was evident by increasing the dose to 0.5 *v*/*v*.

### 2.5. Effect of EO and HL on Juveniles in Soil

**Figure 5:**
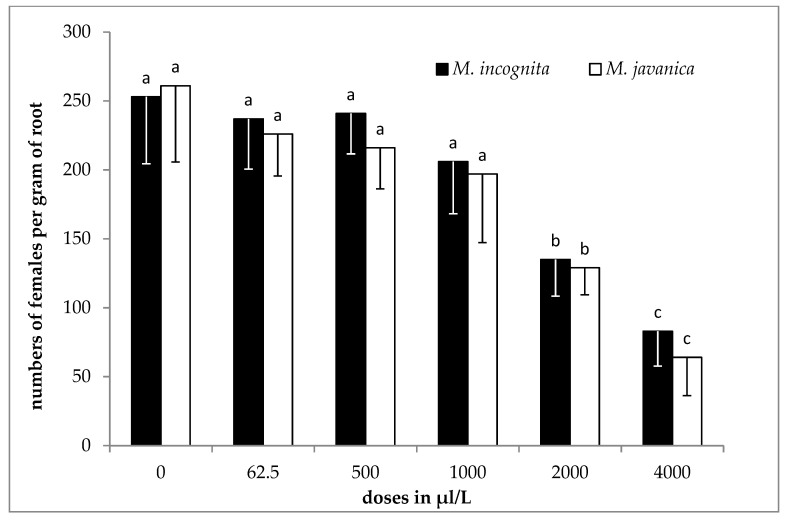
Numbers of females of *M. javanica* and *M. incognita* per gram of root, after transplanting tomato seedlings in soil treated with essential oil of *Satureja hellenica* and inoculating with 600 J2s. Bars with the same color followed by the same letter are not significantly different. Error bars represent the standard deviation of mean.

No significant differences were recorded for the number of females per gram of root, either for *M. incognita* or *M. javanica*, at doses up to 1000 μL/L. Fewer females were recorded as the dose was increased to 2000 μL/L, while a further decrease was recorded by increasing the dose to 4000 μL/L (Figure 5).

**Figure 6:**
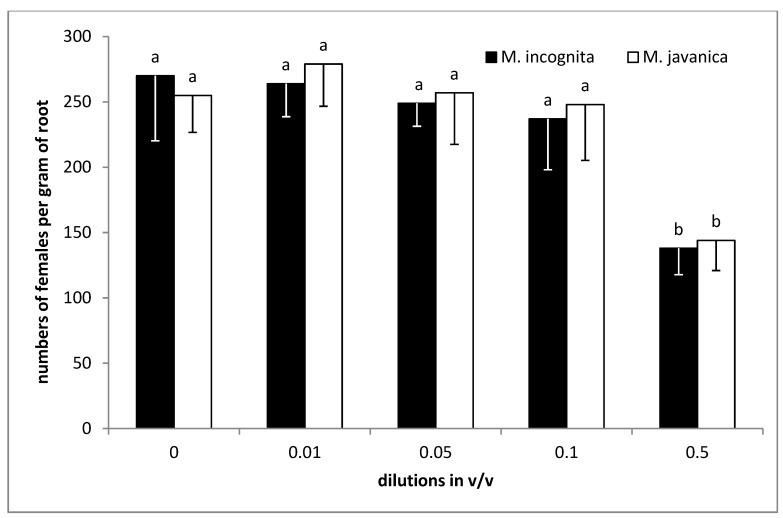
Numbers of females of *M. javanica* and *M. incognita* per gram of root after transplanting tomato seedlings in soil treated with hydrosol of *Satureja hellenica* and inoculating with 600 J2s. Bars with the same color followed by the same letter are not significantly different. Error bars represent the standard deviation of mean.

Additionally, HL at doses up to 0.1 *v*/*v* showed no significant differences compared to the untreated control. A significant decrease was observed after increasing the dose to 0.5 *v*/*v* for both *Meloidogyne* species tested (Figure 6).

## 3. Discussion

This is the first study to show the toxic effect of *Satureja hellenica* extracts against *Meloidogyne incognita* and *M. javanica*. The essential oil caused a strong effect against the different biological stages of either *M. incognita* or *M. javanica*. This study underlines the high nematicidal activity of *S. hellenica* EO against both species tested. A high percentage of paralyzed J2s was recorded after incubating juveniles for 24, 48 and 96 h. The extracts of *Satureja hellenica* seem to have a remarkable nematicidal activity against *Meloidogyne incognita* and *M. javanica*. The percentages over 80% of paralyzed J2s, either at the dose of 2000 μL/L in EO, or the dilution of 0.5 *v*/*v* in hydrosol, after exposure for 48 h suggested potential efficacy from a control point of view. Increased paralyzed J2s were recorded by increasing either the time of exposure (from 24 to 96 h) or the dose of EO. Low doses up to 250 μL/L of EO did not show substantial activity against J2s. However, as the dose was increasing, there was a constant increase in the number of J2s with paralysis. The hydrosol of *S. hellenica* showed lower activity, and J2s paralysis was always inferior to that observed using EO. Substantial paralysis was only recorded after the immersion of J2s in 0.5 *v*/*v* dilution for 48 and 96 h.

*Satureja hellenica* essential oil significantly inhibited egg differentiation. This was noticeable, even at the dose of 250 μL/L, but there was not any further increase of inhibition of *M. incognita* eggs as the dose was increasing. When *M. javanica* eggs were exposed to EO, a high percentage of eggs remained undifferentiated at doses of 500 to 4000 μL/L. Moreover, lower doses of EO (125 and 250 μL/L) have shown a negative effect on egg differentiation. In contrast, the use of *S. hellenica* hydrosol was not that effective in decreasing egg differentiation, for both species. When hydrosol was tested on the differentiation of eggs, a significant decrease was observed only after increasing the dose to 0.5 *v*/*v*. However, at the doses of 0.2 and 0.5 *v*/*v* in hydrosol, there was a non-significant decrease in the hatching of J2s after immersion for 28 days.

Our results show that EO at the two higher doses tested was effective in controlling J2s in soil. Additionally, the hydrosol at the dose of 0.5 *v*/*v* was effective in reducing the numbers of females in roots almost 50% compared to the untreated control. However, in field soil infested by root-knot nematodes, there is a reservoir of inoculum remaining from season to season consisted of either J2s or/and eggs free in soil, or protected inside the gelatinous matrix of the egg sac. It has been reported that nematodes’ eggs consist of three layers, which act as a barrier against nematicides penetration [21]. It is more accurate to use egg hatching than J2s motility experiments [20]. In our experiments, the essential oil inhibited about 85 and 59% of the juveniles from hatching, for *M. incognita* and *M. javanica*, respectively. Our results on hatching inhibition are in agreement with those reported by Barros et al. [22], who worked with EO isolated from *Dysphania ambrosioides* having, as a constituent, *p*-cymene. Additionally, EOs from other plant species, containing *p*-cymene and carvacrol as major constituents, have been shown to inhibit nematode hatching [20,23,24]. When hydrosol was used, the level of juveniles hatched from egg masses (58 and 49% for *M. incognita* and *M. javanica*, respectively) was lower than that observed in EO. The egg hatch inhibition could be due to the ability of either EO’s or HL’s to penetrate egg mass and act on nematode eggs [25].

It is obvious from the results of the present study that there are differences in the ratio of terpenes between the EO and the hydrosol. This could explain the differences of activity between the EO and hydrosol extracted from the same plant species. The essential oil of *S. hellenica* is characterized by both the high presence of monoterpene hydrocarbons (MH) and oxygenated monoterpenes (OM), due mainly to the presence of *p*-cymene, and carvacrol, respectively. The group of MH did not detect the *S. hellenica* hydrosol, while the OM occupied 93.7% of the total composition. The OM exhibited a better extraction to *S. hellenica* hydrosol than the HM, which is explained by the hydrophobicity of the latter. The highest component in the EO is *p*-cymene, which is not present in the hydrosol. On the other hand, the highest constituent of the hydrosol is carvacrol, which is present in EO, but almost 50% lower than that in the hydrosol. Another terpene, borneol, is present in the hydrosol, almost four times more than in EO. Thymol, which is present in EO but not in hydrosol, has been reported in the literature to be active against root-knot nematodes [26]. The components found in *S. hellenica*, either in EO or in hydrosol, are reported to have nematicidal properties, either as constituents of aromatic plant species or as isolated terpenes [25,26]. However, based on our results, it seems that the ratio of each constituent is very important as far as nematicidal effect is concerned. Our results are in agreement with those from Ntalli et al. [27]. They reported that the EO obtained from *O. vulgare* and *O. dictamus* contained 68.5 and 44.3% carvacrol respectively, and had a high nematicidal activity against J2s of *M. incognita*. Laquale et al. [28] reported a strong action of EOs isolated from two Italian ecotypes of Monarda species. Those essential oils had as constituents, among others, *γ*-terpinene and 0-cymene, which are also present in *S. hellenica*. It seems that the presence of carvacrol and *p*-cymene as components of EOs are crucial for their nematicidal activity. However, other substances, even in a lesser quantity, might act synergistically, a fact which should be tested.

## 4. Materials and Methods

### 4.1. Nematode Populations

The populations of *Meloidogyne incognita* and *M. javanica* were collected from infested tomato greenhouses in Heraklion, Crete, and subsequently reared on tomato seedlings (*Solanum lycopersicum* L.) cv. Belladona in a glasshouse in Agricultural University of Athens, Greece. All the seedlings were maintained in plastic pots in controlled conditions (25 ± 2 °C, 16 h light and 8 h dark). The infestation of the seedlings took place when the plants were 6 weeks old, at the four-leaf stage. After 40 days, roots were collected from plants and washed free of soil. Eggs of *Meloidogyne* populations were extracted using 1% sodium hypochlorite solution [29]. Second stage juveniles (J2s) were hatched in a modified Baremann funnel, where eggs were placed. The J2s used in all tests were less than 2 days old.

### 4.2. Plant Material

The plants of *Satureja hellenica* Halásky (Lamiaceae) were collected from the Piges Krias area, near the city of Livadia (Greece). Collected specimens were identified by the fourth author using identification key provided by [30]. The plant material was used to isolate the essential oil and hydrosol used in all trials.

### 4.3. Isolation of Essential Oil and Hydrosol and Determination of Their Chemical Composition

The essential oil was isolated from air-dried leaves and flowers (in the dark, at room temperature) with hydrodistillation (HD), for 3 h in a 5 L Clevenger-type apparatus. The essential oil was dried with anhydrous magnesium sulfate (MgSO_4_). The hydrosol was collected in a separate glass bottle. A part of it was extracted by diethyl ether in order to isolate the volatile compounds. The organic phase was dried over anhydrous magnesium sulfate, filtered, and reduced to a final volume. Both essential oil and organic phase were stored in −18° until their analysis.

The determination of the chemical composition of the plant isolates was achieved by gas chromatography—mass spectrometry (GC-MS) technique [31], using a trace ultra-gas chromatographer coupled with a DSQ II Mass Spectrometer (Thermo Scientific), fitted with a TR-5MS (30 m × 0.25 mm × 0.25 μm) capillary column (Thermo Scientific). The injector and MS transfer line temperatures were set at 220 and 250 °C, respectively. Experimental conditions induced an oven temperature GC programmed from 60 to 250 °C at a rate of 3 °C/min. Helium was used as the carrier gas at a 1 mL/min flow rate. A quantity of 1.0 μL of the diluted samples in the case of EO (1/1000 in acetone, *v*/*v*), or of the organic phase, were injected manually in splitless mode. The MS was operating in EI mode at 70 eV; the ion source temperature was 240 °C, whereas mass spectra were acquired in the scan mode for mass range 35–400. Tentative identification of the compounds based on the comparison of their relative retention indices and mass spectra with corresponding data was reported in the literature’s and instrument’s databases [31]. The relative retention indices (RRI) of compounds were determined with reference to the retention times of (C_8_-C_24_) *n*-alkanes. Relative % percentages of the compounds were obtained electronically from area percentage data.

### 4.4. Effect of EO and Hydrosol on J2s Motility

Cellstar flat bottom 24-well plates were used for all in vitro tests. Solutions of *S. hellenica* essential oil were tested for J2 motility at the doses of 62.5, 125, 250, 500, 1000, 2000 and 4000 μL/L. The essential oil was dissolved in ethanol (Sigma-Aldrich; Italy) and serially diluted in distilled water containing Tween-20, to produce test solutions of the above doses. The ethanol and Tween-20 concentrations (1 and 0.3%, respectively) were tested in preliminary tests, which showed no effect on nematodes. The hydrosol dilutions (in distilled water) tested for J2s motility were 1% (0.01), 2% (0.02), 5% (0.05), 10% (0.1) 20% (0.2) and 50% (0.5) (*v*/*v*). Distilled water was used as a control. Approximately 35 J2s were used per treatment well in the plates. Juveniles’ suspension (0.5 mL) and an equal volume of either EO or HL suspension were pipette into each well. All plates were covered with aluminum foil and incubated at 26 ± 1 °C. J2s observation was conducted using an inverted microscope (100×), after 24, 48 and 96 h incubation time. The juveniles were scored as motile or dead after 10 s observation and robbing their body with a needle. If there was a lack of movement, the J2s were considered to be paralyzed. Each experiment was conducted twice, and every treatment was replicated five times.

### 4.5. Effect of EO and Hydrosol on Egg Differentiation

Eggs of the two species of nematodes were extracted from tomato (*Solanum lycopersicum* cv. Belladona) roots, using the hypochlorite method [29]. The suspension of eggs was placed on a 38 μm sieve, thoroughly rinsed with tap water and collected into a 100 mL beaker. The quantification of eggs suspension was done using an inverted microscope (100×), and the adjusted suspension was used directly in the bioassays.

Essential oil solutions, at the doses of 62.5, 125, 250, 500, 1000, 2000 and 4000 μL/L, and hydrosol dilutions (in distilled water, ethanol and Tween-20) at the concentrations of 0.01, 0.02, 0.05, 0.1, 0.2 and 0.5 *v*/*v*, were tested on the development of eggs. The solutions were prepared as previously described. Distilled water was used as the control. Approximately 50 eggs per well, of which 90% were undifferentiated, were exposed to either EO or HL solutions and incubated at 26 ± 1 °C. Egg suspension (0.5 mL) was pipette into each well, with equal volume of either EO or HL suspension. All plates were covered with aluminum foil to avoid evaporation. For monitoring egg differentiation, eggs were observed on day 0 and day 28, with the aid of an inverted microscope (Zeiss, Germany) at 100× magnification. Eggs were categorized either as differentiated (fully developed juvenile) or undifferentiated (containing only cells). The experiment was conducted twice and each treatment was replicated five times.

### 4.6. Hatching Inhibition as Affected by the Presence of EO and Hydrosol

Mature egg masses of *Meloidogyne incognita* and *M. javanica* were handpicked using sterilized forceps from roots free of soil. Egg masses were placed in small plastic extraction trays made by six cm petri dishes. Solutions of *S. hellenica* essential oil (62.5, 125, 250, 500, 1000 and 2000 μL/L) or hydrosol (0.01, 0.02, 0.05, 0.1, 0.2 and 0.5 *v*/*v*), initially dissolved in ethanol and brought to volume using Tween-20 in water, were added to each extracting tray to cover egg masses. Egg masses were maintained for seven days, and then test solutions were removed by washing them with tap water, and placed in new extracting trays filled with clean water. Extracting trays were covered with aluminum foil to avoid evaporation and placed in an incubator at 26 ± 1 °C. Hatched J2s were counted every seven days, they were discarded, and the water was substituted with fresh one. The experiment was terminated when J2s did not emerge any longer, 35 days later. Then, the egg masses were picked from Petri dishes, placed in a drop of water on a glass microscope slide, gently squashed between the slide and coverslip, and the number of unhatched eggs per egg mass was counted under an inverted microscope. The experiment was conducted twice and each treatment was replicated five times.

### 4.7. Effect of EO and Hydrosol on Juveniles in Soil

The efficacy of *S. hellenica* EO and HL against *M. javanica* were evaluated using tomato seedlings, cv Belladonna. Seedlings at the four-leaf stage grown in soil were collected from a greenhouse at Gargaliani village, Peloponnese. Soil was autoclaved to kill any plant parasitic nematodes. Essential oil solutions, at the doses of 62.5, 500, 1000, and 4000 μL/L, and hydrosol dilutions (in distilled water) at the concentration of 0.01, 0.05, 0.1, and 0.5 *v*/*v*, were tested on J2s in soil. Five plastic pots were filled with the treated soil, and 1 mL of a suspension containing 500 J2s was used for inoculation. Pots were covered with aluminum foil to avoid water evaporation and maintained at 25 ± 1 °C for 24 h to make sure that juveniles came into contact with the chemicals. Then, a tomato seedling (cv Belladonna) at the four-leaf stage was transplanted in the center of each pot. All plants were placed in a growth room at 25 ± 1 °C and 28 days later were uprooted, stems were removed, and roots were gently washed free of soil. Roots were stained using acid fuchsin, as described in Byrd et al. [32] Roots were then washed in water and placed in vials containing equal volumes of glycerol and distilled water. Female nematodes were counted in the whole root system of each plant using a stereoscopic microscope at 12.5× magnification. All treatments were replicated five times, while the experiment was conducted twice.

### 4.8. Statistical Analysis

A one-way analysis of variance (ANOVA) was performed using the general linear model (GLM) in SAS (SAS University Edition). Treatments’ means were compared using the Tukey’s HSD test at *p* < 0.05. In case no variation was revealed, the data from two experiments were combined and analyzed together.

## 5. Conclusions

In conclusion, our work showed that *S. hellenica* extracts can control *M. incognita* and *M. javanica*. Our results underlined the paralysis activity against J2s, along with the inhibition of egg differentiation and hatching. However, further experimentation is necessary for the determination of the most efficient rate and dose of these extracts.

## Figures and Tables

**Table 1:** Chemical composition of the essential oil and hydrosol of *Satureja hellenica*.

No	RRI *	Compound	*S. hellenica* e.o.	*S. hellenica* Hydrosol
1	940	*α*-Pinene	1.5	- ^#^
2	948	Camphene	1.3	-
3	979	1-Octen-3-ol	-	1.6
4	981	*β*-Myrcene	1.0	-
5	999	3-Octanol	-	0.3
6	1014	*α*-Terpinene	1.1	-
7	1024	*p*-Cymene	27.5	-
8	1057	*γ*-Terpinene	4.6	-
9	1075	*cis*-Sabinene hydrate	2.4	4.6
10	1083	Terpinolene	0.4	-
11	1082	*cis*-Linalool oxide	-	0.7
12	1100	Linalool	0.6	0.7
13	1102	*trans*-Sabinene hydrate	0.4	2.5
14	1136	1-Terpineol	0.3	-
15	1143	*cis*-2-Pinanol	0.6	1.3
16	1152	Camphor	0.5	1.2
17	1179	Borneol	6.8	20.4
18	1183	4-Terpineol	3.6	6.7
19	1188	*p*-Cymen-8-ol	0.3	2.8
20	1195	*α*-Terpineol	0.6	1.9
21	1204	*cis*-Dihydrocarvone	0.2	-
22	1223	Coahuilensol methyl ether	-	0.2
23	1232	Thymol methylether	1.4	-
24	1243	Carvacrol methylether	6.8	0.5
25	1270	Geranial	0.1	-
26	1292	Thymol	0.3	-
27	1299	Carvacrol	23.3	50.1
28	1348	Thymol acetate	-	0.3
29	1354	Eugenol	-	0.2
30	1418	(*Ε*)-Caryophyllene	3.5	0.2
31	1438	Aromadendrene	0.4	-
32	1457	*α*-Humulene	0.2	-
33	1491	Viridiflorene	0.2	
34	1495	Bicyclogermacrene	0.3	
35	1507	*β*-Bisabolene	3.5	-
36	1575	Spathulenol	1.4	0.4
37	1580	Caryophyllene oxide	2.7	0.3
38	1652	*α*-Eudesmol	0.2	0.1
		Total (%)	98.0	97.0
		Monoterpene Hydrocarbons (MH)	37.4	-
		Oxygenated Monoterpenes (OM)	48.2	93.7
		Sesquiterpene Hydrocarbons (SH)	8.1	0.2
		Oxygenated Sesquiterpenes (OS)	4.3	0.8
		Others	-	2.3

* RRI: Relative Retention Index obtained on TR-5MS column using a series of *n*-alkanes (C8–C24). ^#^ Not detected.

**Table 2:** Effect of essential oil on the motility of *M. incgonita* second stage juveniles (J2s) after immersion in test solutions, at the doses of 0, 62.5, 125, 250, 500, 1000, 2000 and 4000 μL/L, for 24, 48 and 96 h.

Dose (μL/L)	Exposure Time (h)		
	24	48	96
	Dead J2s (%)	Dead J2s (%)	Dead J2s (%)
0	0.0 d	2.6 d	10.4 e
62.5	0.0 d	2.0 d	25.3 d
125	0.0 d	4.3 d	25.7 d
250	0.5 d	7.5 d	21.4 d
500	1.1 d	30.6 c	56.6 c
1000	7.7 c	38.5 c	85.6 b
2000	31.1 b	81.2 b	100 a
4000	99.1 a	100 a	100 a

**Table 3:** Effect of essential oil on the motility of *M. javanica* J2s after immersion in test solutions at the doses of 0, 62.5, 125, 250, 500, 1000, 2000 and 4000 μL/L for 24, 48 and 96 h.

Dose (μL/L)	Exposure Time (h)		
	24	48	96
	Dead J2s (%)	Dead J2s (%)	Dead J2s (%)
0	0.3 d	1.1 d	12.4 d
62.5	3.7 d	6.9 cd	23.2 cd
125	3.6 d	5.2 cd	31.5c
250	2.4 d	7.0 c	22.7 cd
500	4.6 d	13.1 c	46.2 c
1000	12.7 c	35.4 b	82.5 b
2000	48.5 b	86.2 a	100 a
4000	90.1 a	98.9 a	100 a

**Table 4:** Effect of hydrosol on the motility of *M. incognita* J2s after immersion in test solutions at the dilutions 0, 0.01, 0.02, 0.05, 0.1, 0.2 and 0.5 *v*/*v* for 24, 48 and 96 h.

Dilution (*v*/*v*)	Exposure Time (h)		
	24	48	96
	Dead J2s (%)	Dead J2s (%)	Dead J2s (%)
0	0.0 b	0.5 b	1.9 c
0.01	0.6 b	1.7 b	5.2 bc
0.02	0.5 b	2.6 b	7.7 b
0.05	1.0 b	1.9 b	11.6 b
0.1	0.8 b	1.2 b	8.3 b
0.2	0.2 b	1.0 b	8.3 b
0.5	13.1 a	75.9 a	93.3 a

**Table 5:** Effect of hydrosol on the motility of *M. javanica* J2s after immersion in test solutions at the dilutions 0, 0.01, 0.02, 0.05, 0.1, 0.2 and 0.5 *v*/*v*, for 24, 48 and 96 h.

Dilution (*v*/*v*)	Exposure Time (h)		
	24	48	96
	Dead J2s (%)	Dead J2s (%)	Dead J2s (%)
0	0.3 b	0.8 b	6.5 c
0.01	0.9 b	2.0 b	14.0 bc
0.02	0.8 b	3.0 b	18.4 b
0.05	1.0 b	1.6 b	13.7 bc
0.1	1.4 b	2.4 b	22.8 b
0.2	2.5 b	6.8 b	28.8 b
0.5	46.1 a	79.7 a	96.4 a
